# Evaluation Protocol Sensitivity in Frequency-Constrained Truss Optimization: A Comparative Study of Adaptive and Evolutionary Metaheuristics

**DOI:** 10.3390/biomimetics11040235

**Published:** 2026-04-02

**Authors:** Ahmad Ghiaskar, Farid Taheri

**Affiliations:** Advanced Composites and Mechanics Laboratory, Department of Mechanical Engineering, Dalhousie University, Halifax, NS B3H 4R2, Canada; ghiaskar@dal.ca

**Keywords:** frequency-constrained truss optimization, polar fox algorithm, evaluation protocol sensitivity, metaheuristic algorithms, structural optimization, benchmarking bias

## Abstract

Metaheuristic algorithm benchmarking in frequency-constrained truss optimization (TPFC) has been shown to be sensitive to evaluation protocols, yet this sensitivity has not been systematically investigated. This study addresses this gap by introducing a dual protocol benchmarking framework to quantify protocol-induced ranking bias across five metaheuristic algorithms, including the Polar Fox Algorithm, NSM-LSHADE-CnEpSin, NSM-LSHADE-SPACMA, NSM-MadDE, and NSM-BO. The algorithms are evaluated on five frequency-constrained truss benchmarks with 10, 37, 52, 72, and 200 bars, under two FE-based protocols with MaxFEs of 10,000 × D and 20,000 × D. Under the standardized protocol, LSHADE variants achieve the highest rankings, while the Polar Fox Algorithm ranks lowest despite demonstrating strong early-stage search efficiency, primarily due to its non-uniform FE allocation. Under the extended protocol, the performance gap of the Polar Fox Algorithm is reduced by 44 to 79 percent, with rankings improving to second and third, while ranking reversals are observed on larger-scale problems. These findings show that algorithmic rankings in TPFC optimization are strongly dependent on evaluation protocols rather than reflecting intrinsic algorithmic quality. Furthermore, fixed FE ceilings are shown to disadvantage adaptive and exploration-intensive algorithms. The proposed dual-protocol framework provides a generalizable basis for protocol-aware benchmarking, revealing the conditions under which FE-based comparisons may systematically favor uniform-allocation methods in constrained structural optimization.

## 1. Introduction

Optimization of truss structures is one of the most studied areas due to their vast applications in multiple fields such as civil, mechanical, offshore, and aerospace engineering [1,2]. In addition to their geometry, material properties, and static performance requirements, the optimization of structural systems with consideration of their dynamic response and frequency-dependent characteristics is gaining increased attention, especially from the perspective of their safety and serviceability [3,4]. Note that most traditional structural optimization examples have been considered under static conditions (e.g., stress, displacement, and weight constraints) [5]. Therefore, optimization of truss problems considering frequency constraints (TPFCs) has been gaining attention recently [6]. Such a class of problems is more complex in nature than its classical counterparts [7]. To avoid resonance in any structural system, the natural frequencies of a structure must be kept away from the excitation frequency induced by various loadings (e.g., wind, seismic activities, traffic activities, etc.) [8,9].

The nature of TPFCs also causes the associated mathematical model to involve high levels of complexity and features, which significantly differentiate it from the formulation of typical static structural optimization problems [10]. For example, the frequency constraints involved in TPFCs involve eigenvalue solutions. This is due to the extremely nonlinear features involved, which lead to a non-convex, multimodal solution space with several local minima [11]. In trusses, frequency constraints are also affected by geometric and cross-sectional configurations, material distribution of truss structures, and their associated objective and constraint functions, thus rendering them as non-convex and discontinuous, with a complex feasible set [6,12]. These parameters, in turn, make gradient-based methods generally ineffective in solving TPFCs. Advanced structural optimization frameworks have also addressed related challenges in nonlinear and reliability-based contexts, including elasto-plastic truss design under geometric nonlinearity [13] and thermo-mechanical reliability-based topology optimization under material uncertainties [14].

The desire to find optimal or near-optimal solutions to complex engineering optimization problems has also created a need for advanced computational methods that can efficiently search for solutions in irregular solution spaces without requiring gradient information [15]. The answers to real-world optimization problems are often limited due to the increasing complexity of engineering design and manufacturing problems, which has led to the development of several optimization methods using traditional mathematical approaches [16,17]. In the pre-metaheuristic research era, mathematical optimization was the primary method for seeking solutions. However, because mathematical techniques are more well-defined and deterministic in nature, they are often ineffective for most real-world situations, which are frequently characterized by the presence of multiple local optima and complex interactions among constraints [18].

Moreover, nature-inspired Metaheuristic Optimization (MHO) algorithms are a class of highly efficient optimization algorithms that solve problems by imitating biological phenomena or physical processes found in nature [19,20]. MHO algorithms are gradient-free, highly flexible, and possess a robust mechanism for escaping local optima, making them a popular and suitable tool for solving complex optimization problems in various engineering fields [21]. The researchers’ motivation to design MHO algorithms continues to grow, particularly for achieving appropriate solutions in complex engineering problems and sophisticated optimization scenarios such as image processing and frequency-constrained structural design [22]. Beyond structural design, MHO algorithms such as PFA have also demonstrated strong performance in composite materials optimization, including maximizing impact energy absorption of fiber-reinforced composites subject to manufacturing and geometric constraints [23]. MHO algorithms are typically categorized into several types, including swarm-based, biogeographical, physics-based, evolutionary, and others [24,25]. Each class is effective for a set of problems with a compatible nature [26,27].

Over the past few years, animal behavior, particularly in terms of how different species interact with their environment to solve problems, has become a significant source of inspiration for researchers across various fields [28]. For instance, Nemati et al. [29] employed the Connected Banking System Optimization (CBSO) algorithm to optimize the sizing of truss structures and successfully demonstrated the viability of their novel algorithm by tackling several benchmark truss structures (i.e., 10-, 17-, 18-, 25-, 72-, and 120-bar trusses). Avcı et al. [30] conducted a detailed analysis of the sizing and layout optimization of benchmark truss systems using the Improved Stochastic Ranking Evolution Strategy (ISRES), which yielded competitive results for various instances. Yokota et al. [31] presented a procedure for solving optimal weight design problems of 10-bar truss structures using the Genetic Algorithm (GA). They laid down the basic methodology for solving such a problem through evolutionary algorithms.

The predator-based and survival-based MHO algorithms are the most recent additions to the family of nature-inspired algorithms. Öztürk and Kahraman [32] presented a comprehensive comparative study on MHO search algorithms for truss optimization, offering a unique overview of the stability and complexity analyses of these algorithms while ranking LSHADE-EpSin, LSHADE-CnEpSin, SHADE, and LSHADE among the best-performing algorithms. Kaveh [33] has been investigating metaheuristic algorithms for optimal structure design for several years. He has made several contributions to the development of optimization methods, including chaos-embedded and collision-based optimization approaches. Azizi et al. [34] employed Chaos Game Optimization (CGO) to optimize the shape and size of truss structures subject to frequency constraints, demonstrating the algorithm’s effectiveness on various benchmark problems, including truss systems with 10, 37, 52, 72, and 120 bars. Kaveh and Mahjoubi [35] presented a Hypotrochoid Spiral Optimization approach for sizing and layout optimization of truss systems under multiple frequency constraints, demonstrating enhanced convergence behavior of their proposed algorithm.

Recent comprehensive studies on truss optimization have reported challenges related to inconsistent experimental settings, insufficient algorithmic comparisons, and the absence of stability analysis in optimization processes. Öztürk and Kahraman [32] identified that, while truss problems are among the most studied real-world engineering design problems, their optimization presents significant issues. These challenges include conducting studies across varying experimental settings, inconsistent performance comparisons among the algorithms, and a lack of investigation into the algorithms’ stability and computational complexity. Consequently, researchers continue to test metaheuristic search algorithms in frequency-constrained truss problems, while also investigating their stability in reaching optimal solutions. Öztürk and Kahraman [32] have recently proposed four improved evolutionary algorithms. They observed that, among a large number of competitive algorithms, only a small number of them have superior performance in terms of convergence and robustness, and LSHADE-EpSin [36], LSHADE-CnEpSin [37], SHADE [38], LSHADE-SPACMA [39], and GSK [38] algorithms have shown very good convergence and stability properties. It is worth noting that the search space poses several challenges for frequency-constrained problems, and very few algorithms can find the optimal solution in a reasonable time.

While prior works such as Öztürk and Kahraman [32] have acknowledged inconsistencies in experimental settings, none have systematically isolated and quantified the effect of FE-budget size on algorithmic rankings in TPFC problems. Specifically, no prior study has demonstrated that ranking reversals, not merely marginal score differences, can arise solely from the choice of termination criterion. Furthermore, the mechanism by which adaptive, non-uniform FE-allocation strategies are structurally penalized under fixed FE ceilings has not been previously characterized or measured. The present dual-protocol framework addresses this gap, and its logic extends to any constrained structural optimization context involving algorithms with heterogeneous per-iteration computational costs.

The mentioned complexities demand robust optimization algorithms that can effectively handle multiple constraints while ensuring computational efficiency and solution reliability. The benchmark suite comprises the well-known 10-, 37-, 52-, 72-, and 200-bar truss systems, which have been extensively studied under static conditions; here, they are considered under frequency constraints. The frequency-constrained optimization benchmarks will include geometry and constraint patterns that several researchers have widely employed [40,41,42,43,44]. These problems have been used as testbeds to evaluate the performance of various algorithms across different problem scales and constraint levels; therefore, they will demonstrate a comprehensive analysis of the PFA’s ability to cope with the complexity and non-uniformity of the frequency constraints and the solution space.

It should be noted that the main reason for employing the Polar Fox Algorithm is that it has been supported by several successful records on dealing with complex search spaces, while it also retains a balance between exploitation and exploration [45]. PFA has a more effective search operator that is inspired and derived from how polar foxes hunt their prey, thus enabling the algorithm to be adaptive in dealing with multimodal search spaces, as is the case in frequency-constrained optimization problems [33]. It should be noted that Öztürk and Kahraman [32,46] also identified the NSM-LSHADE-CnEpSin as one of the most capable in the LSHADE family of algorithms [38] for optimizing truss systems. Therefore, a specific comparison is made between the Polar Fox Algorithm and NSM-LSHADE-CnEpSin to demonstrate stability and achieve a high success rate on frequency-constrained problems across small-, medium-, and large-scale truss systems.

This study evaluates the Polar Fox Algorithm alongside four established metaheuristics (LSHADE-CnEpSin, LSHADE-SPACMA, MadDE, and Butterfly Optimization) on five frequency-constrained truss benchmarks (10-bar, 37-bar, 52-bar, 72-bar, and 200-bar) under two complementary protocols: (i) FE-based (MaxFEs = 10,000 × D) for direct comparison with state-of-the-art results [46] and (ii) extended FE-based (MaxFEs = 20,000 × D) to evaluate budget sensitivity and deeper refinement. This protocol framework addresses protocol-induced bias in metaheuristic assessment, revealing how termination criteria and FE budgets systematically influence algorithmic rankings in frequency-constrained structural optimization.

## 2. Related Works

Frequency-constrained truss optimization has gained significant attention over the last decade. Several researchers have proposed various MHO approaches for tackling complex computational issues in these problems [47]. In this section, recent works on TPFC optimization are surveyed, focusing on trends in algorithms, performance, and research approaches to gain insight into the current state-of-the-art and to situate the present work within that context.

**Figure 1:**
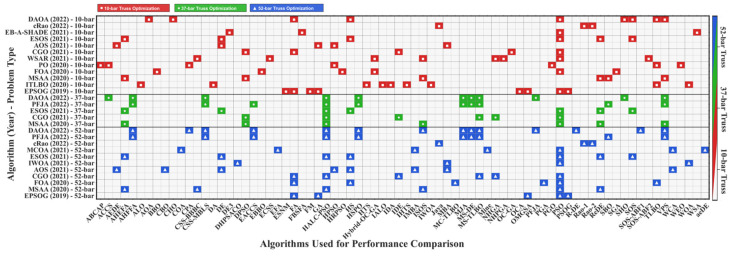
Algorithm comparison heatmap in TPFC optimization. Proposed algorithms: DAOA [60], cRao [57], EB-A-SHADE [73], ESOS [69], AOS [68], CGO [34], WSAR [67], PO [70], FOA [71], and MSAA versus benchmark methods across 10-bar, 37-bar, and 52-bar truss problems.

In recent years, most studies have focused on these three specific truss topologies, i.e., 10-, 37-, and 52-bar trusses [48,49]. Larger-scale benchmarks such as the 72-bar and 200-bar trusses have received comparatively limited attention in the literature. The selection of three truss structures with varying complexities, ranging from the relatively simple 10-bar planar truss to the more complex 52-bar space truss, together with the two 72-bar and 200-bar trusses, more carefully considers the challenges of algorithm evaluation. These serve as the required test beds for comparing algorithmic performance [50]. It should be noted that the visualization in Figure 1 is restricted to the three classical benchmark problems (10-, 37-, and 52-bar), reflecting the current coverage of the literature rather than the full range of problem scales considered in the present study.

The computational methods used in previous studies have undergone significant evolution over the past few years. In the early works, only traditional algorithms such as the GA [51], PSO [52], and DE [53] were employed. In recent years, research interest in more advanced variants or hybrid approaches (i.e., those tailored to solve the complex eigenvalue problems that appear in frequency-constrained structural optimization) has grown significantly [54]. For example, different modifications of differential evolution with improved particle swarm strategies and new nature-inspired algorithms suitable for structural optimization have been developed and applied [38].

The 10-bar frequency-constrained truss problem has been a base case study for a vast array of optimization algorithms. Several researchers have examined algorithms from the traditional form (Jaya [55] and Rao [56] variants) to more complex algorithms (Enhanced Rao (eRao) [57], Firefly-based algorithms (FBSFA) [58], and Enhanced SHADE variants [59]).

The 37-bar truss structure has also attracted considerable attention due to its intermediate complexity and practical relevance in structural engineering applications [47]. Such studies have particularly focused on algorithms such as the Dynamic Arithmetic Optimization Algorithm (DAOA) [60], Jaya algorithm (PFJA) [61], Enhanced Sum of Squares (ESOS) [62], and various Teaching–Learning-Based Optimization (TLBO) variants [63,64].

The most difficult of the optimization problems mentioned is the 52-bar frequency-constrained truss problem, which has been used as a test case to motivate and examine the development of more efficient optimization methods [47]. Some works on this include the Modified Cuckoo Optimization Algorithm (MCOA) [65], Improved Whale Optimization Algorithm (IWOA) [66], Advanced Optimization Strategy (AOS), and several hybrid variants of other methods [22].

Recent research efforts in TPFC optimization have demonstrated significant algorithmic diversity and innovation across the three benchmark problems. Notable contributions include DAOA introduced by Khodadadi et al. [60] for natural frequency constraints and the WSAR algorithm developed by Baykasoğlu et al. [67] for multiple frequency constraints. Azizi et al. [68] applied AOS considering discrete design variables, while Nguyen-Van et al. [69] proposed ESOS for multi-constraint optimization. Azizi et al. [34] also utilized CGO for shape and size optimization, whereas Awad [70] implemented the PO algorithm, and Liu et al. [71] enhanced FOA with an adaptive vision search strategy.

The visualization in Figure 1 provides a comprehensive overview of the algorithms implemented for the three main frequency-constrained truss optimization problems. Color coding indicates the number of applications, reflecting the research status and activity in the related studies. Overall, well-known algorithms such as PSO [52], GA [51], and DE [72] have been used in more than 60% of past studies. It should be noted that, among the various algorithms, the DAOA [60] has been considered in most past studies for examining the three truss case studies; however, its application to other case studies has been comparatively limited. The development of algorithms has become more sophisticated over time (i.e., between 2019 and 2022), with hybrid algorithms accounting for a large percentage of the recently presented algorithms. It should also be noted that, to the best of the authors’ knowledge, the 37-bar truss problem has been used in only five studies, compared with the 10- and 52-bar truss cases, which have been examined in 12 and 11 studies, respectively.

Upon analyzing recent works in this area, one identifies a set of crucial methodological issues. For instance, the inconsistencies in the experimental conditions and evaluation frameworks have resulted in difficulty in concluding a fair performance comparison [46]. The limited number of competitive algorithms available also confines the evaluation to a subset of methods, thus leading to an incomplete performance analysis [12,74,75,76,77,78].

**Figure 2:**
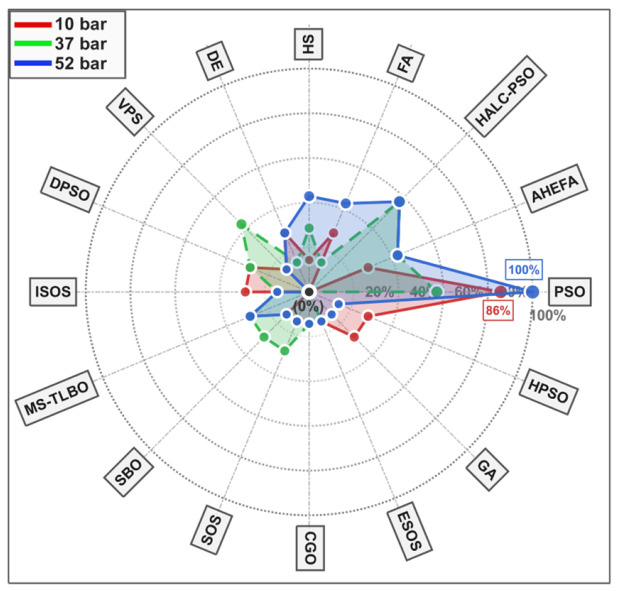
Radar chart illustrating research intensity and algorithmic adoption patterns across 10-, 37-, and 52-bar frequency-constrained truss problems.

The radar chart in Figure 2 presents a multidimensional visualization of algorithmic adoption trends relative to the three benchmark problems noted earlier. The illustration shows the dominance of swarm intelligence approaches (specifically, their PSO variant) with usage rates exceeding 80% in the 10-bar truss, as evolutionary algorithms have been adopted in 60–70% of the studies [67,73]. As also seen, nature-inspired algorithms exhibit varying levels of adoption, with the greatest penetration in the 52-bar truss case study (45%), compared to its simpler counterpart configurations [79,80], indicating the research community’s preference for population-based approaches when considering challenging frequency-constrained problems.

Statistically, swarm intelligence and evolutionary algorithms exhibit the best adaptability to frequency-constrained problems [6,52]. As stated previously, hybrid approaches have gained more attention recently, owing to the fact that a single-strategy algorithm cannot always solve frequency-constrained problems [81]. The most recent advancements in evolutionary frequency planning are adaptive parameter control, problem-dependent knowledge, and machine-learning methods [82,83]. However, no prior work has systematically examined how evaluation protocol choices, specifically termination criteria and FE budgets, influence algorithmic rankings, meaning that reported performance advantages may reflect protocol compatibility rather than intrinsic algorithmic quality.

Performance evaluation methodologies have been continuously evolving, with more rigorous statistical analysis procedures enhancing their scientific rigor. However, issues remain to be addressed, such as standardizing benchmarks and evaluation criteria to enable fair comparisons across different research endeavors. It is noticeable that: (1) there is a scarcity of comprehensive comparative studies that encompass a wide range of methodologies; (2) the focus has often been on algorithm development rather than systematic performance analysis; and (3) the aspects of stability analysis and success rate evaluation have not been given adequate attention.

A critical examination of these prior works reveals three consistently unresolved issues. First, while experimental inconsistencies are acknowledged, no study has systematically isolated and quantified how termination criteria alone, independent of algorithmic quality, alter rankings. Second, the structural disadvantage imposed on adaptive, non-uniform FE allocation strategies by fixed FE ceilings has not been identified or measured. Third, all existing TPFC benchmarking studies rely on a single evaluation protocol, preventing any assessment of how sensitive conclusions are to that choice. These gaps collectively motivate the dual protocol framework adopted in the present study. Specifically, this study interrogates the implicit assumption that FE-based protocols ensure inherent fairness when comparing algorithms with inherently different per-iteration computational costs; the dual-protocol framework is designed to expose, rather than resolve, this structural asymmetry.

The specific methodological contributions of this study are threefold: (i) the first systematic quantification of protocol-induced ranking bias in TPFC optimization, demonstrating that ranking reversals emerge solely from termination criterion choice; (ii) identification of the mechanistic cause of this bias, whereby fixed FE ceilings structurally disadvantage adaptive non-uniform FE-allocation strategies regardless of their intrinsic quality; and (iii) proposal of a dual-protocol benchmarking principle as a generalizable framework for fair algorithm assessment wherever competing methods differ in per-iteration FE consumption.

Drawing from the insights gathered and the gaps in research that have been observed from this literature review, especially regarding the uneven coverage of the three benchmark problems in existing studies, as well as the incomplete evaluation of various algorithms, the present research centers on the comprehensive and methodical assessment of the Polar Fox Algorithm and four reference algorithms across the three classical benchmark truss configurations (10, 37, and 52 bar) and further extends the evaluation to larger-scale 72-bar and 200-bar trusses to address the existing gaps in the literature. The study further adopts standardized FE-based and extended-budget protocols to enable direct comparison with the state-of-the-art benchmark [46] and to systematically quantify the protocol-induced bias inherent in such comparisons. Not only does this focus rectify the previously noted methodological issues but it also advances efforts toward more stringent standards for algorithm evaluation within the TPFC optimization community.

## 3. Algorithmic Framework for Frequency-Constrained Truss Optimization

Frequency-constrained truss optimization (TPFC) problems typically involve many design variables, nonlinear eigenvalue constraints, and highly nonconvex feasible regions. Effective and robust solution strategies, therefore, require optimization methods capable of navigating multimodal landscapes while maintaining feasibility with respect to strict dynamic constraints.

In this study, the Polar Fox Algorithm (PFA) [27] is employed as one of the five compared metaheuristic optimizers, alongside the four Natural Survivor Method (NSM)-based algorithms recently identified by Öztürk and Kahraman [46] as the most competitive methods for TPFC problems. The PFA, inspired by the adaptive survival behavior of Arctic foxes, provides a stochastic search framework suitable for complex structural optimization tasks.

All experiments are performed over 30 independent runs per algorithm per problem using MATLAB R2025a (MathWorks (Natick, MA, USA) on an Intel Core i7 workstation (3.6 GHz, 16 GB RAM). All algorithms are initialized using uniform random sampling within the prescribed design variable bounds, with MaxFEs as the sole stopping criterion. Algorithm configurations follow their original publications: NSM-LSHADE-CnEpSin and NSM-LSHADE-SPACMA use a population size of D × 18 with arc_rate = 1.4, memory size = 5, and *p* = 0.11; NSM-MadDE employs an adaptive population ranging from 4 to 2 × D^2^ with arc_rate = 2.3 and *p* = 0.18; NSM-BO uses a population of 30 with scab = 1.3, scsb = 1.4, and rcpp = 0.0036; PFA uses a population of 40 [27] with parameters as defined in [37,46]. All NSM-based algorithms incorporate the NSM update strategy as described in [46].

This unified framework allows a fair comparison between PFA and the four NSM-based algorithms MadDE [84], LSHADE-CnEpSin, LSHADE-SPACMA [39], and BO [85], which were reported as top-performing TPFC solvers out of 152 tested metaheuristics in the benchmark study of Öztürk and Kahraman [46].

### 3.1. Polar Fox Algorithm (PFA)

The Polar Fox Algorithm (PFA) [27] is a population-based metaheuristic inspired by the hunting strategy and social organization of Arctic foxes. The algorithm uses four complementary behavioral steps: experience-based exploration, leader exploitation, adaptive mutation, and fatigue-induced diversification. This framework is designed to guide search in complex optimization landscapes and is applied in this study to solve frequency-constrained truss problems.

#### 3.1.1. Population Initialization and Leash Formation

The initial population of polar foxes represents potential solutions distributed throughout the feasible design space. The population matrix X is defined as [27]:

(1) $$x=\left[\begin{array}{ccc} \begin{array}{cc} x_{1}^{1} & x_{2}^{1}\\ x_{1}^{2} & x_{2}^{1} \end{array} & \begin{array}{c} \ldots \\ \ldots \end{array} & \begin{array}{c} x_{d}^{1}\\ x_{d}^{d} \end{array}\\ \begin{array}{cc} \vdots & \vdots \end{array} & \ddots & \vdots \\ \begin{array}{cc} x_{1}^{k} & x_{2}^{k} \end{array} & \ldots & x_{d}^{k} \end{array}\right]$$

where each polar fox position is initialized using:

(2) $$x^{i}=LB+\vec{r_{1}}.(UB-LB)$$

where $LB=[lb_{1},\;lb_{2}\ldots .lb_{d}]$ and $UB=[ub_{1},\;ub_{2}\ldots .ub_{d}]$ represent the lower and upper bounds, respectively, $\vec{r_{1}}$ is a random vector with elements in [0, 1], k is the population size, and d is the dimensionality.

#### 3.1.2. Behavioral Grouping

The population is partitioned into four groups (G1, G2, G3, G4) with distinct behavioral matrices [PF, LF, a, b, m]. Group weights are updated dynamically [27]:

(3) $$W_{i}^{new}=W_{i}+\frac{t^{2}}{{NG}_{i}}$$

where t is the iteration count and ${NG}_{i}$ is the group size.

#### 3.1.3. Experience-Based Phase

Individuals perform exploratory jumps based on historical success:

(4) $$x^{i}\left(t+1\right)=x^{i}\left(t\right)+P.D$$

(5) $$P=\vec{r_{2}}.PF_{i}\;{PF}_{i}=PF.a^{\left(z-1\right)}$$

(6) $$D=cos(\vec{r_{3}})$$

where z counts phase repetitions, $\vec{r_{2}}\in [0,1]$, and $\vec{r_{3}}\in [0{}^{\circ},180]$. The phase terminates when [27]:

(7) $$\left\{\begin{array}{c} PF_{i}<m.PF\\ \;f\left(t\right)<f(t-1) \end{array}\right.$$

#### 3.1.4. Leader-Based Phase

Foxes converge toward the best solution L:

(8) $$x^{i}\left(t+1\right)=x^{i}\left(t\right)+\vec{r_{4}}.\left(x^{i}\left(t\right)-L\right).LF_{i})$$

(9) $$LF_{i}=LF.b^{y-1}$$

where $\vec{r_{4}}\in [-1,1]$ and y counts repetitions. Termination:

(10) $$\left\{\begin{array}{c} LF_{i}<m.LF\\ \;f\left(t\right)<f(t-1) \end{array}\right.$$

#### 3.1.5. Leader Motivation and Mutation

When stagnation is detected via:

(11) $$Crit-Cond=\left(NLM>MLM\right)or(t>0.8\times NI)$$

behavioral matrices reset and NM solutions mutate [27]:

(12) $$NM=\left\{\begin{array}{c} NP-1,\;\;\;\;\;\;\;\;\;if\;critical\\ MF\times NP,\;\;\;\;\;otherwise \end{array}\right.$$

(13) $$x^{i}=LB+\vec{r_{5}}.\left(UB-LB\right),i=[1\;2\ldots .NM]$$

#### 3.1.6. Fatigue Simulation

Group energies decay after each iteration:

(14) $$G_{j}=min(G_{j}-G_{jr},\;G_{ji}),\;\;j\;\in \;\{1,\;2,\;3,\;4\}$$

Groups shrinking below 10% of NP are restored to $G_{j}m.$

#### 3.1.7. Computational Complexity

The computation time of PFA is influenced by the nested iterative structure of experience-based and leader-based phases, population-wide behavioral strategy application, and fitness evaluation at each iteration. Time Complexity $O(n^{2})$, where n is the problem dimensionality, attributed to the double loop structure (z iterations in the experience phase and y iterations in the leader phase for each NP individual) and eigenvalue decomposition for frequency constraint evaluation. The complete list of notations and parameters used in the PFA mathematical formulation is presented in the reference [27]. In summary, the experience-based phase (Equations (4)–(7)) drives global exploration through adaptive jumps, while the leader-based phase (Equations (8)–(10)) enforces local exploitation toward the best solution. The mutation and fatigue mechanisms (Equations (11)–(14)) prevent stagnation by periodically diversifying the population. This architecture inherently allocates multiple FEs per iteration, distinguishing PFA from DE-based methods and directly motivating the dual-protocol evaluation framework adopted in this study.

### 3.2. Adaptation to Frequency-Constrained Engineering Problems

#### 3.2.1. Mathematical Problem Formulation

The frequency-constrained truss optimization problems involve two categories of design variables: cross-sectional areas of structural members (A) and nodal coordinates (∆) for geometry optimization. The optimization aims to minimize the total structural mass while meeting natural frequency requirements and satisfying variable bounds.

The general mathematical formulation is expressed as [32,33,86]:

Minimize:

(15) $$f\left(x\right)=\sum_{i=1}^{n}\rho .A_{i}.L_{i}\;\mathrm{where}\;x=(A,\;∆)$$

Subject to:

(16) $$g_{i}\left(x\right)=\frac{\omega_{j}^{min}}{\omega_{j}(x)}-1\leq 0\;\;\;\;\;\;\;\;\;j=1,2,\ldots ,m$$

(17) $$A_{k}^{min}\leq A_{k}\leq A_{k}^{max}\;\;\;\;\;\;\;\;\;\;\;\;\;\;\;k=1,2,\ldots ,p\;$$

(18) $${∆}_{s}^{min}\leq {∆}_{s}\leq {∆}_{s}^{max}\;\;\;\;\;\;\;\;\;\;\;\;\;\;\;s=1,2,\ldots ,r\;\;$$

where $L_{i}=\sqrt{{\left(x_{2}-x_{1}\right)}^{2}+{\left(y_{2}-y_{1}\right)}^{2}+{\left(z_{2}-z_{1}\right)}^{2}}$ represents the length of the element i, $\omega_{j}(x)$ denotes the $j^{th}$ natural frequency as a function of design variables, and m, p, r represent the number of frequency constraints, cross-sectional variables, and geometric variables, respectively [87].

The following assumptions are adopted in this study: (i) all truss members are linearly elastic with isotropic material properties; (ii) member buckling and geometric nonlinearity are neglected; (iii) non-structural masses are lumped at the nodes; (iv) damping effects are excluded from the eigenvalue analysis; and (v) all design variables are treated as continuous. These assumptions are consistent with those adopted in the reference benchmark studies [32].

#### 3.2.2. Eigenvalue Analysis Integration

The natural frequencies required for constraint evaluation are obtained through standard generalized eigenvalue analysis of the structural system:

(19) $$\left(K\left(x\right)-\omega_{j}^{2}M\left(x\right)\right)\phi_{j}=0$$

where $K\left(x\right)$ and $M\left(x\right)$ are the design-dependent global stiffness and mass matrices, respectively, and $\phi_{j}$ represents the $j^{th}$ mode shape vector.

For truss structures, the consistent element mass matrix in local coordinates is formulated as [32,46]:

(20) $$M_{e}=\frac{\rho A_{e}L_{e}}{6}\left[\begin{array}{cc} {2I}_{3} & I_{3}\\ I_{3} & {2I}_{3} \end{array}\right]$$

The transformation to global coordinates employs the direction cosine matrix $T_{e}$:

(21) $$M_{e}^{global}=T_{e}^{T}M_{e}^{local}T_{e}$$

Concentrated masses are incorporated by direct addition to the diagonal terms of the global mass matrix:

(22) $$M_{ii}=M_{ii}+m_{concentrated,i}\;I_{3}$$

Several numerical challenges arise in the eigenvalue analysis of frequency-constrained trusses. Mode switching, in which the order of eigenvalues changes across design iterations, can cause discontinuities in constraint gradients and adversely affect convergence. Eigenvalue sensitivity to cross-sectional area variations is inherently high in slender members, which amplifies numerical instability near constraint boundaries. Furthermore, repeated eigenvalues introduce non-differentiability in the constraint functions, making gradient-based approaches unreliable. The computational cost of eigenvalue decomposition scales as O(n^3^) with the number of structural degrees of freedom, making it the dominant cost per function evaluation and directly relevant to the FE budget analysis presented in Section 3.3.

In large-scale problems such as the 200-bar truss, the O(n^3^) eigenvalue cost becomes the dominant computational factor per function evaluation, further underlining the practical significance of the FE budget analysis and the evaluation protocol adopted in this study.

#### 3.2.3. Constraint Handling and Penalty Function Strategy

PFA incorporates an exterior penalty function approach to transform the constrained optimization problem into an unconstrained formulation:

(23) $$\Phi \left(x\right)=f\left(x\right)+P(x)$$

The penalty function is defined as:

(24) $$P\left(x\right)=\gamma \sum_{j=i}^{m}max{\left(0,g_{i}\left(x\right)\right)}^{2}$$

where $\gamma =10^{8}$ is selected as the penalty parameter to ensure adequate constraint enforcement while maintaining numerical stability. The quadratic penalty formulation provides smooth gradient behavior near constraint boundaries or structural optimization problems.

#### 3.2.4. Modal Frequency Analysis and Constraint Evaluation

The natural frequencies are obtained from the generalized eigenvalue problem

(25) $$K\phi_{i}=\lambda_{i}M\phi_{i}\;\;\;\;\;\;\;\;\;\;\;\;\;\;\;\;\;\;\;\;\;\omega_{i}=\sqrt{\lambda_{i}},\;\;\;\;\;\;\;\;\;\;\;\;\;\;\;\;\;\;\;\;\;\;f_{i}=\frac{\omega_{i}}{2\pi }$$

Frequency constraints are formulated in a general form as

(26) $$\;f_{i}\geq f_{i}^{min},\;\;\;\;\;\;\;\;\;\;f_{j}\leq f_{j}^{max},$$

depending on whether a lower- or upper-bound requirement is specified. The exact numerical bounds for each benchmark problem (37-bar planar truss and 52-bar dome truss) are given in Section 4 [88].

This general formulation is applied in Section 4 to the 37-bar and 52-bar benchmark trusses, where problem-specific frequency limits are specified.

### 3.3. Critical Note on Evaluation Protocols

The Polar Fox Algorithm employs a dual-phase search mechanism (experience-based exploration and leader-based intensification, Equations (6) and (9)) that allocates multiple function evaluations (FEs) per iteration through iterative refinement cycles. While this adaptive resource allocation enables thorough local search, it results in higher FE consumption per iteration compared to single-evaluation methods. Öztürk and Kahraman [46] demonstrated that iteration-based termination introduces systematic bias because algorithms consume FEs at vastly different rates and proposed the standardized FE-based limit MaxFEs = 10,000 × D to ensure equal computational budgets across methods. However, equal budget does not imply equivalent search processes; algorithms with high per-iteration FE consumption necessarily complete fewer search iterations within the same budget, introducing a search granularity asymmetry that this study explicitly investigates rather than resolves.

However, this FE ceiling presents a fundamental trade-off: algorithms with non-uniform FE allocation may terminate after significantly fewer iterations, potentially preventing completion of their refinement mechanisms before exhausting the FE budget. To provide a comprehensive assessment, this study adopts two complementary protocols: (i) FE-based protocol at MaxFEs = 10,000 × D, strictly replicating the benchmark framework from [46] to enable direct comparison with four leading algorithms (LSHADE-CnEpSin, LSHADE-SPACMA, MadDE, and BO) across 30 independent runs, and (ii) extended FE-based protocol at MaxFEs = 20,000 × D to investigate budget sensitivity and scalability on larger problems. The value of 20,000 × D was selected as the minimum extension sufficient to permit completion of PFA’s dual-phase exploration–intensification cycles, which are prematurely terminated under the 10,000 × D ceiling due to non-uniform FE consumption per iteration; doubling the budget provides adequate search depth while remaining computationally tractable. This multi-protocol framework ensures results are benchmark-comparable while revealing how termination criteria influence algorithmic assessment and intrinsic convergence capabilities under sufficient search depth.

Although demonstrated here for TPFC benchmarks, the protocol dependence hypothesis is generalizable to other constrained engineering optimization domains, such as topology and shape optimization, wherever competing algorithms exhibit non-uniform FE consumption per iteration.

## 4. Engineering Design Problems

### 4.1. Frequency-Constrained 10-Bar Truss

**Figure 3:**
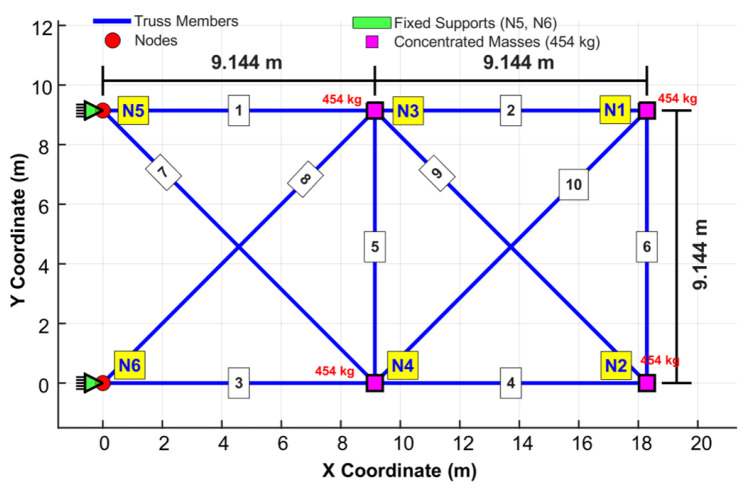
Configuration of the frequency-constrained 10-bar planar truss system.

The 10-bar planar truss optimization problem addresses the size optimization of a symmetric planar truss with 10 members connected by five nodes, with the objective of minimizing the total structural weight under natural frequency constraints [33]. The topology of the 10-bar planar truss system is depicted in Figure 3, with nodes 5 and 6 rigidly supported and additional masses added at the free nodes. This problem involves 10 design variables representing cross-sectional areas (0.645–12.9 cm^2^) and three frequency constraints for the first three modes, using material properties of an elastic modulus of 68,950 MPa and a density of 2770 kg/m^3^, thereby effectively testing an algorithm’s ability to account for the dynamic response of a given system.

### 4.2. Frequency-Constrained 37-Bar Truss

**Figure 4:**
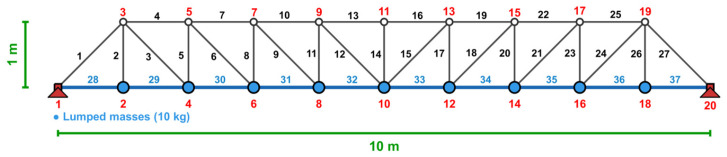
Configuration of the symmetric 37-bar planar truss system (Joint numbers in red, member numbers in black and lumped massess are identified by blue circles).

The simply supported 37-bar planar truss is also a classical benchmark problem for simultaneous size and geometry optimization with frequency constraints, as shown in Figure 4, with the same minimization objectives as the 10-bar truss. This problem effectively evaluates the performance of the metaheuristic algorithms for optimizing mixed-variable structural systems.

Table 1 summarizes the design of variable groups and bounds. Table 2 shows the frequency constraints and material properties. There are 19 design variables in this optimization, 14 sizing variables for the symmetric member groups and 5 geometric variables for the Y-coordinates of the upper chord nodes with symmetric pairs.

The objective function minimizes the total structural weight: $W=\sum_{i=1}^{37}\rho A_{i}L_{i}$ subject to frequency constraints that ensure adequate dynamic performance. The problem is a suitable case study for testing the performance of optimization algorithms applied to mixed variable types and nonlinear frequency.

### 4.3. Frequency-Constrained 52-Bar Dome Truss

**Figure 5:**
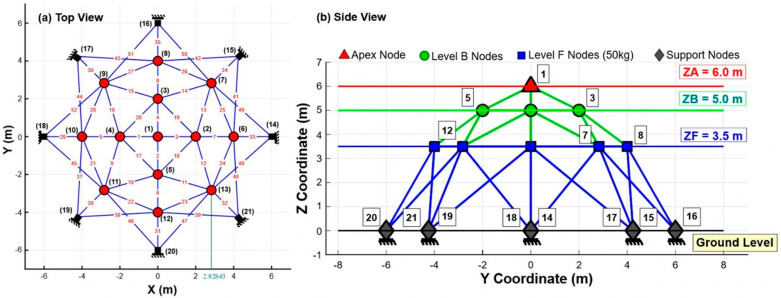
Schematic of the 52-bar dome truss: (**a**) top view showing node layout and member connectivity, (**b**) side view of elevation profile with level designations.

The 52-bar dome truss system is a 3D simultaneous size and topography optimization problem with frequency constraints [33]. This dome structure, as illustrated in Figure 5, involves optimizing both the members’ cross-sectional areas and the nodal coordinates as design variables. The structure comprises four levels, the apex node (ZA), intermediate level B (ZB), lower-level F (ZF), and ground-level supports, characterized by a few geometric parameters that determine the spatial configuration. A non-structural mass of 50 kg is attached to all free nodes, and, due to structural symmetry, the 52 members are grouped into eight design variables as detailed in Table 3.

This structural design optimization problem includes 13 design variables (8 sizing + 5 layout) with constraints on the first two natural frequencies of the structure, providing a challenging three-dimensional optimization test case for algorithm evaluation.

### 4.4. Large-Scale Truss Optimization Benchmarks

**Figure 6:**
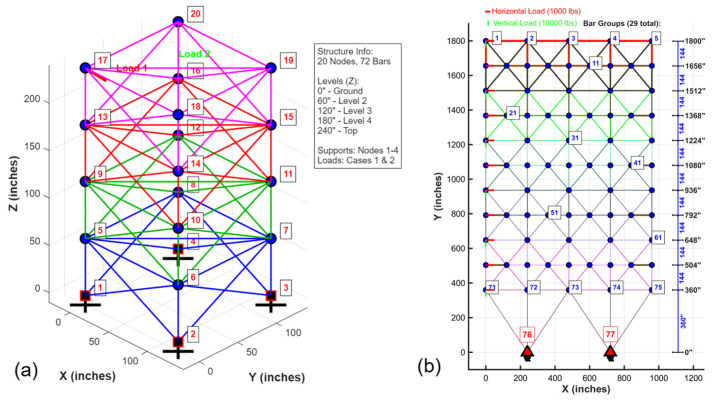
Large-scale truss problems: (**a**) 72-bar spatial truss; (**b**) 200-bar planar truss.

To assess more comprehensively the performance characteristics of the proposed algorithms for large-scale structural optimization, two well-known large-scale truss benchmark problems, namely the 72-bar spatial truss [89] and the 200-bar planar truss [90], as illustrated in Figure 6a,b, respectively, are considered. The 72-bar spatial truss consists of 72 members and 20 joints, forming a three-dimensional (3D) structural system with five levels, pin-supported joints at the four bottom corner nodes, and a concentrated load applied at the top joint. Owing to structural symmetry, the members are grouped into 16 sizing design variables, and the optimization objective is to minimize the total structural weight subject to stress and displacement constraints. The 200-bar planar truss comprises 200 members and 29 sizing design variables, forming a two-dimensional (2D) structural system with pinned joints, and is subjected to multiple loading conditions, including horizontal, vertical, and combined loads. In this benchmark problem, the structural weight is minimized while satisfying stress constraints under all loading conditions. The key information regarding element groupings, material properties, and boundary conditions for both truss structures is provided in Table 4.

## 5. Results and Discussion

As noted in the Critical Note on Evaluation Protocols, evaluation protocols critically affect algorithmic assessment. To ensure benchmark-comparable and comprehensive evaluation, two complementary experiments were conducted: (i) standardized tests under the 10,000 × D FE budget, following recent TPFC benchmarks, and (ii) extended budget evaluation under 20,000 × D to assess protocol sensitivity and scalability. This study first reports results from the first protocol, based on the 10,000 × D ceiling, across 30 runs to enable a direct comparison of the performance of the five algorithms (Section 5.1). Subsequently, Section 5.2 presents extended evaluations under the 20,000 × D budget, examining both scalability on larger benchmarks (72-bar and 200-bar trusses) and systematic budget sensitivity analysis across all five problems.

### 5.1. Algorithmic Performance Analysis

#### 5.1.1. Convergence Behavior

**Figure 7:**
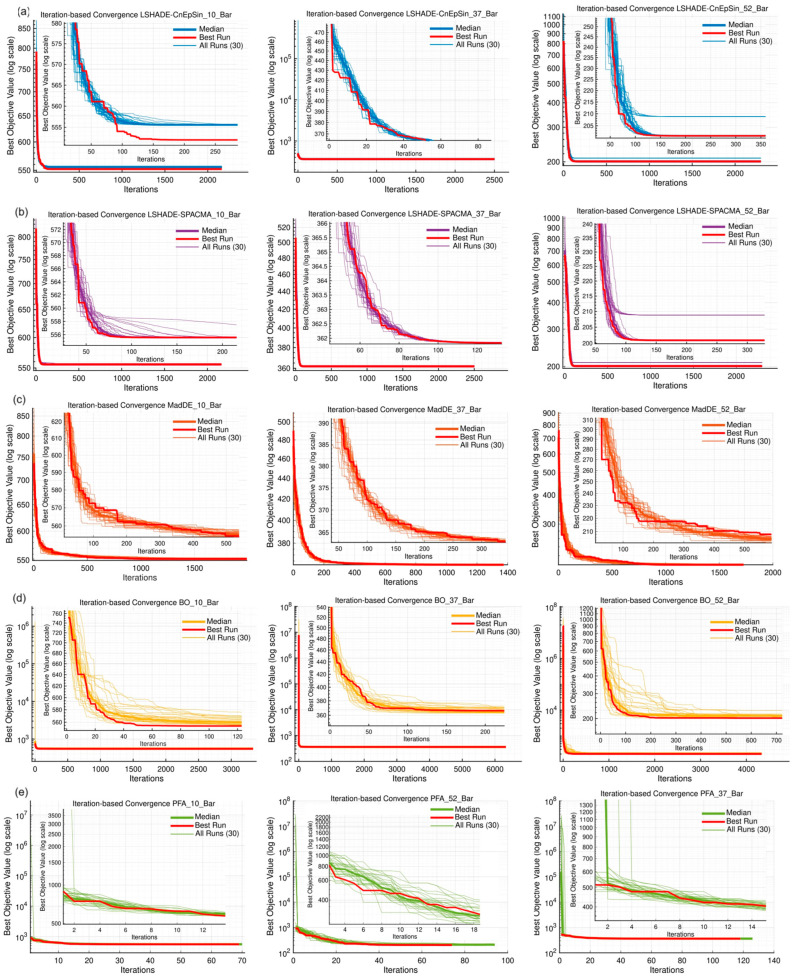
(**a**–**e**) Iteration-based convergence curves (median, best run, and all runs) for five algorithms on the 10-, 37-, and 52-bar truss problems.

The convergence dynamics of the five algorithms were first examined under the standardized FE-based protocol (10,000 × D), as recommended in recent TPFC benchmarks. Figure 7a–e shows the iteration-based trajectories across 30 runs for the 10-, 37-, and 52-bar truss problems. LSHADE-CnEpSin and LSHADE-SPACMA algorithms consistently converged to the known optima with minimal variance. MadDE also showed robust behavior, albeit with slower rates, while BO exhibited rapid early progress but higher dispersion. PFA, in contrast, produced sharp initial improvements but quickly plateaued at higher objective values, reflecting its exploration–intensification mechanism: aggressive early search coupled with uneven FE allocation.

This early stagnation is mechanistically attributed to PFA’s experience-based phase (Equations (4)–(7)), which consumes a disproportionately large number of FEs per iteration through iterative refinement cycles, leaving insufficient budget for the leader-based intensification phase (Equations (8)–(10)) to converge under the 10,000 × D ceiling.

The runtime results reported in Table 5 further support the above trends, where PFA required the least computational time (≈approximately 16 s for the 10-bar, 139 s for the 37-bar, and 115 s for the 52-bar), reflecting its lower iteration counts compared to DE-based competitors. For the 10-bar truss, MadDE achieved the best overall ranking (1.67) with a best cost of 552.0422, essentially matching the known optimum. LSHADE-CnEpSin followed closely (rank 2.00, best 552.0420), while PFA trailed with an average rank of 4.33 and a best cost of 552.4025, thus approximately 0.06% higher than the leading methods. In the 37-bar problem, LSHADE-CnEpSin (1.33) and LSHADE-SPACMA (1.67) both reached the exact optimum of 361.8358 across all runs, whereas PFA produced a best cost of 362.1815, corresponding to an error of about 0.095% and ranking last (4.67). Similarly, for the 52-bar truss, MadDE (1.67) and LSHADE-CnEpSin (2.00) attained best costs of 200.9190 and 200.9163, respectively, while PFA remained weakest (5.00) with a best of 202.3339, roughly 0.7% above the optimum. These results confirm that, although the dual-phase search mechanism of PFA enables rapid early descent, it fails to refine solutions to the same precision as DE-based competitors under the FE ceiling, a limitation exacerbated by its very low average iteration counts (62, 111, and 83 for the 10-, 37-, and 52-bar problems, respectively).

#### 5.1.2. FE-Based Convergence and Efficiency

**Figure 8:**
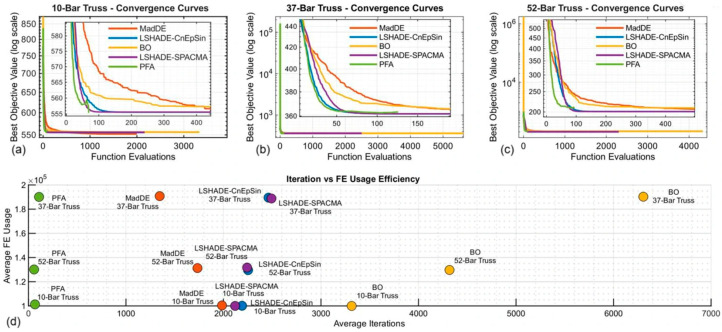
(**a**–**c**) FE-based convergence curves and (**d**) efficiency scatter plots of algorithms on the three truss benchmarks.

Furthermore, FE-based convergence curves were generated to account for FE allocation directly. Figure 8a–c exhibits that LSHADE variants and MadDE approach optimal solutions smoothly, while PFA stalls above the optimum, despite its rapid initial descent. The efficiency scatter seen in Figure 8d highlights the imbalance; PFA consumed a disproportionately large number of FEs per iteration, yielding very low iteration counts but high FE usage. This imbalance is also reflected in the runtimes shown in Table 5, where PFA’s shorter iteration cycles result in faster execution, albeit at the cost of less refined solutions, consistent with the evaluation protocol concerns outlined in the Critical Note on Evaluation Protocols, while penalizing adaptive strategies such as PFA that rely on early exploration phases.

#### 5.1.3. Stability and Convergence Rates

**Figure 9:**
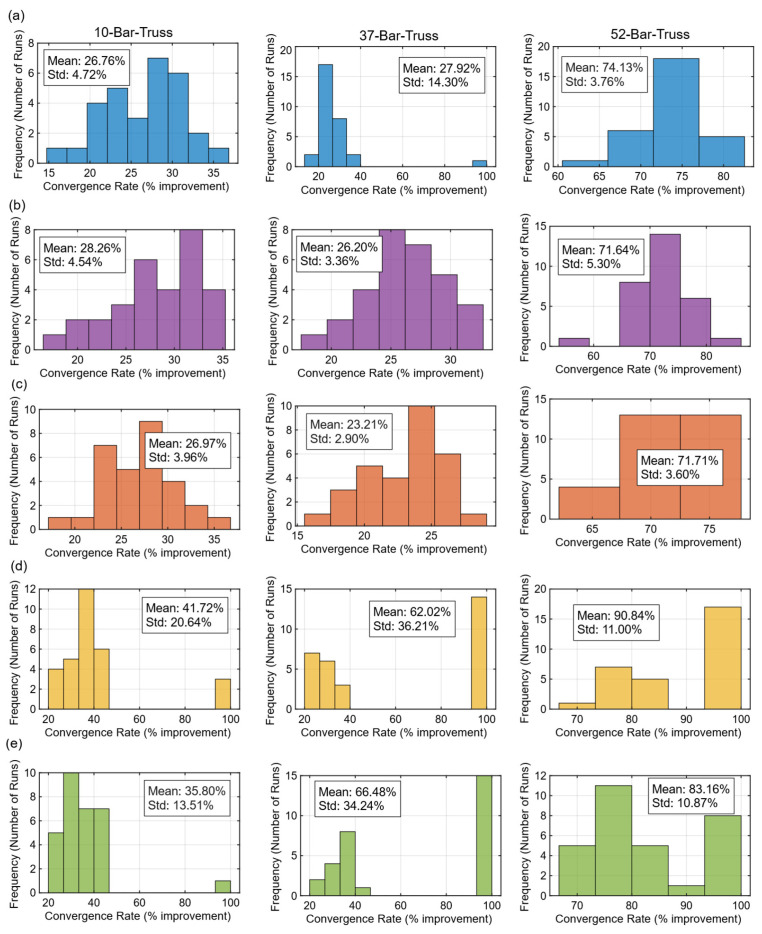
Convergence rate distributions across 30 runs for each algorithm on the 10-, 37-, and 52-bar trusses: (**a**) LSHADE-CnEpSin; (**b**) LSHADE-SPACMA; (**c**) MadDE; (**d**) BO; (**e**) PFA.

Stability was further analyzed through convergence rate distributions. Figure 9a–e shows that LSHADE-CnEpSin and LSHADE-SPACMA achieved narrow, low-variance distributions, confirming their robustness. MadDE was similarly consistent, while BO produced higher mean gains but with very large variance. PFA displayed the widest spreads, with some runs achieving 90% improvement, while many others stagnated, resulting in standard deviations exceeding 30% on the 37-bar problem. These patterns reflect the algorithm’s adaptive search strategy; intensive early exploration can yield high payoffs in some cases, but outcomes remain unstable under limited computational budgets.

#### 5.1.4. Reliability and Efficiency

**Figure 10:**
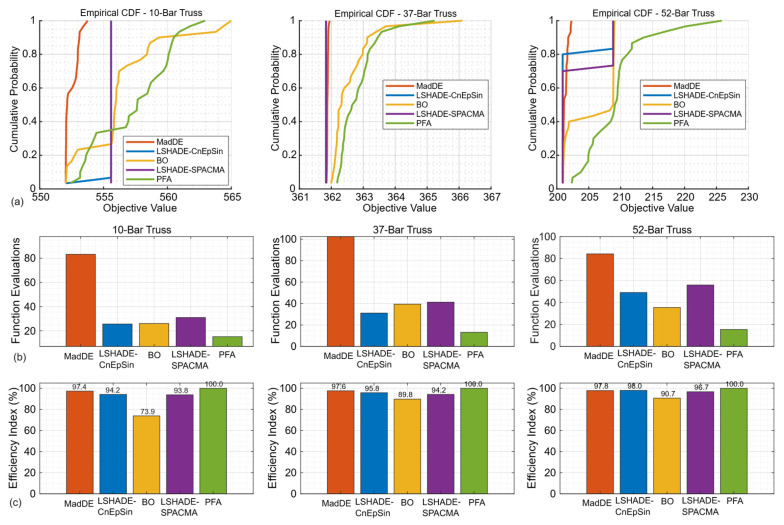
Empirical Performance comparison across the 10-, 37-, and 52-bar trusses: (**a**) empirical CDFs of best objec-tive values; (**b**) function evaluations required to reach 90% convergence; (**c**) normalized efficiency index for each algorithm.

The cumulative distribution functions (CDFs) of best costs (Figure 10a) further confirm the reliability gap; DE-based methods dominated the probability of near-optimal performance, while PFA lagged across all problems. Efficiency indicators tell a more nuanced story. As shown in Figure 10b, PFA consistently required the fewest FEs to reach 90% improvement, demonstrating its strength in early search. This was reflected in the normalized Efficiency Index, where PFA scored 100% in all cases (Figure 10c). However, this “efficiency” largely captures early progress rather than consistency in final performance, again illustrating the trade-off imposed by the FE-based stopping rule. Importantly, this trade-off is mirrored in execution time; while PFA consistently achieved the shortest runtimes, DE-based algorithms maintained superior reliability despite longer computational costs.

#### 5.1.5. Comparison of the Convergence Rates

**Figure 11:**
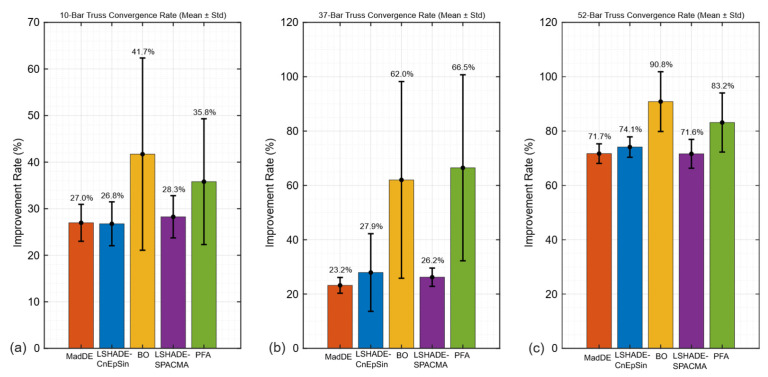
Mean convergence rates with standard deviations, comparing algorithmic stability on the 10-, 37-, and 52-bar trusses:: (**a**) 10-bar truss; (**b**) 37-bar truss; (**c**) 52-bar truss.

Finally, the aggregated convergence rates with error bars (Figure 11) highlight the differences among the algorithms. LSHADE-CnEpSin and LSHADE-SPACMA offered moderate but highly consistent rates, MadDE was stable with lower values, while BO was highly variable. PFA combined high average rates (up to 83% in the 52-bar case) with large variability, reinforcing that its adaptive resource allocation strategy leads to unbalanced outcomes under the standardized FE ceiling.

Overall, the standardized protocol confirms the dominance of DE-based methods in terms of stability and reliability, while PFA remains at a disadvantage due to its adaptive exploration–intensification strategy, which unevenly consumes FEs and yields low iteration counts. These results signify the importance of evaluation protocols in shaping perceptions of competitiveness.

### 5.2. Extended Computational Budget Analysis

To determine whether the rankings observed in Section 5.1 reflect intrinsic algorithmic quality or protocol-induced bias, all five benchmarks are re-evaluated under an extended budget of MaxFEs = 20,000 × D.

Table 6 presents a comprehensive budget sensitivity analysis comparing results under two computational budgets across all five benchmarks. For the three classical problems (10-, 37-, and 52-bar trusses), the 10,000 × D values reproduce Table 5 results (30 runs), while the 20,000 × D outcomes are derived from 20 independent runs on problems 10 through 200. The results reveal striking patterns; across all three classical benchmarks, PFA’s best costs improved by 0.16 to 1.04 kg (reductions of 0.029 to 0.51%), while LSHADE-CnEpSin exhibited negligible changes (less than 0.001 kg), as its refinement capacity had already saturated under the lower budget. This differential response directly reflects the resource allocation strategies of these mechanisms: LSHADE exhausts the budget uniformly across iterations (approximately 180 FEs per iteration), enabling complete refinement cycles even under tight constraints; in contrast, PFA’s adaptive dual-phase approach allocates FEs non-uniformly based on search-phase requirements and benefits substantially from extended budgets that permit completion of exploration–intensification cycles. Consequently, PFA’s performance gap relative to LSHADE-CnEpSin decreased by 44–79%, and its rankings improved from 4th–5th to 2nd–3rd, confirming that the 10,000 × D protocol introduces systematic bias against adaptive resource allocation strategies.

**Figure 12:**
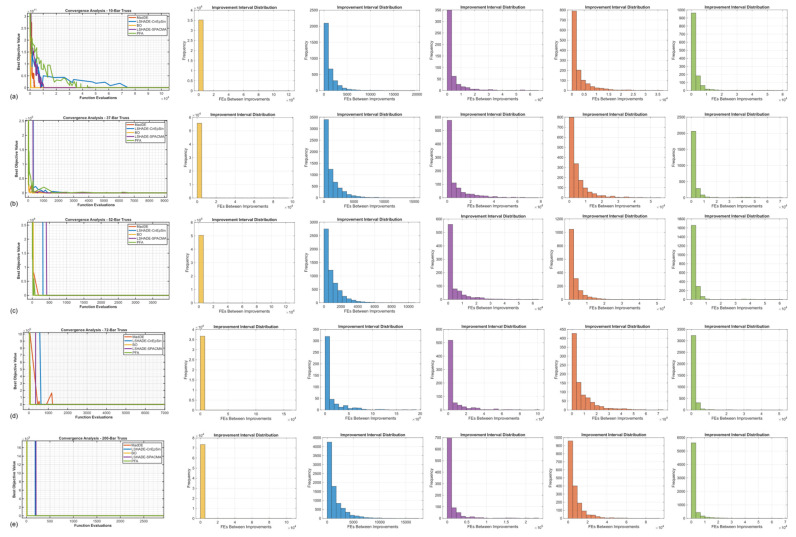
Convergence trajectories and solution interval distributions for larger benchmarks under extended budget (MaxFEs = 20,000 × D): (**a**) 10-bar truss; (**b**) 37-bar truss; (**c**) 52-bar truss; (**d**) 72-bar truss; (**e**) 200-bar truss.

Figure 12 illustrates the convergence trajectories and improvement interval distributions for all benchmarks under the 20,000 × D protocol. Across the classical problems, LSHADE-CnEpSin exhibits a remarkably stable convergence pattern while achieving the best mean results; this is further evidenced by its histograms, which show a dense, clustered distribution of improvement intervals in the early stages. Notably, within the 20,000 × D budget, PFA demonstrates a significant performance leap compared to the results in Section 5.1. Its convergence curves—particularly for the 10- and 37-bar trusses—reveal a striking speed in locating the optimal region with minimal function evaluations.

In the larger benchmarks, the analysis in Figure 12d confirms the absolute dominance of the BO algorithm on the 72-bar truss, achieving the optimum with a minimum standard deviation of 0.300 kg. Finally, the 200-bar problem (Figure 12e) shows late-stage, highly precise convergence among the top-tier methods. In this case, the final solution clustering is exceptionally tight; the zero-standard deviation for LSHADE-CnEpSin and values below 0.5 kg for BO and PFA in the table align perfectly with the high frequency of intervals concentrated near the origin in the final column of histograms.

**Figure 13:**
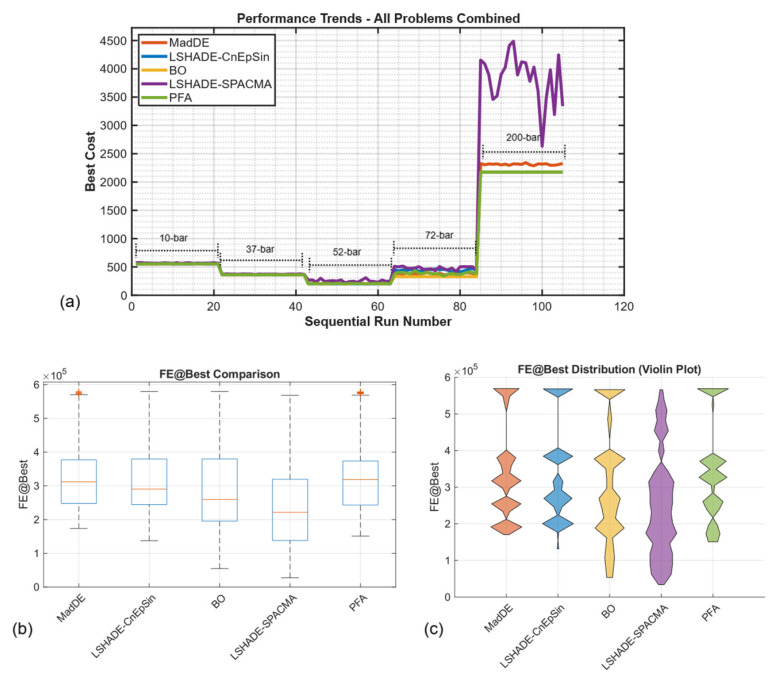
Performance trends and FE allocation patterns under extended computational budget (MaxFEs = 20,000 × D): (**a**) sequential performance across all five benchmarks: best costs from sequential runs; (**b**,**c**) fundamental differences in resource allocation strategies.

The reference study [46] examined the 10,000 × D protocol with the aim of ensuring “equal computational budgets” among algorithms, arguing that FE-based limits provide fairer comparisons than iteration counts. While this logic is valid for methods with uniform per-iteration FE consumption (such as LSHADE, which averages approximately 180 FEs per iteration), it inadvertently disadvantages adaptive strategies like PFA, which allocate resources non-uniformly based on search-phase requirements. Figure 13a visualizes this disparity, showing sequential runs for five problems and five algorithms under 20,000 × D; it can be observed that LSHADE-CnEpSin uniformly maintains low costs across all problem segments, while PFA exhibits competitive performance on the 10-, 37-, and 52-bar problems and ranks second on the 72-bar segment. The FE@Best distributions (Figure 13b,c) also demonstrate that LSHADE typically consumes more than 95% of its budget before reaching optimal solutions, whereas PFA achieves best costs earlier but lacks remaining FEs for final refinement under the 10,000 × D ceiling.

For the 72-bar and 200-bar trusses, which were not previously evaluated at 10,000 × D in this study, the 20,000 × D results reveal problem-dependent ranking shifts. The 72-bar truss exhibits an anomalous pattern; PFA achieved 341.03 kg (rank 2) and outperformed both LSHADE variants (407.92 and 414.56 kg, ranks 4 and 5), while BO dominated at 328.24 kg. This reversal, in which LSHADE-CnEpSin held first rank across all Section 5.1 problems but shows fourth here, suggests that intermediate-scale problems favor exploration–intensive mechanisms when adequate search depth is provided. The 72-bar spatial truss has only four design variables (D = 4). This results in a relatively low-dimensional but highly nonconvex search space with many local optima due to the three-dimensional geometry of the spatial truss structure. In such spaces, LSHADE’s strategy of equal FE allocation over iterations leads to premature convergence to local optima because the exploitation strategy dominates. On the other hand, PFA and BO are more likely to escape local optima if provided with sufficient FEs, as they are more focused on exploration. This specific characteristic of the 72-bar spatial truss problem highlights the importance of exploration over refinement. Conversely, the 200-bar truss restored more conventional rankings: LSHADE-CnEpSin (2174.33 kg, rank 1), BO (2174.39 kg, rank 2), and PFA (2174.60 kg, rank 3, deviation 0.012%). The very tight clustering within a 0.27 kg range across 29 dimensions, comparable to the optimal solutions identified in reference [46], demonstrates that, under extended budgets, distinctions among top-tier methods become marginal rather than fundamental.

This cumulative evidence substantiates the protocol-dependence hypothesis: termination criteria fundamentally shape algorithmic rankings in TPFC optimization. FE-capped protocols favor methods with uniform per-iteration resource consumption (LSHADE and MadDE) by enabling maximization of iteration counts, while systematically disadvantaging adaptive strategies that allocate FEs non-uniformly based on search-phase requirements. This bias—which was not addressed in the original benchmark framework [46] and its exclusive focus on single-protocol evaluation—can obscure methods that would perform better under alternative termination criteria. Extended budgets partially neutralize this imbalance and reveal that algorithms penalized under FE ceilings can demonstrate competitive solution quality when afforded adequate search depth. These findings advocate multi-protocol benchmarking practices; reporting results under both FE-based and iteration-based termination criteria provides a more complete assessment of algorithmic capabilities, particularly for methods employing adaptive mechanisms that trade iteration counts for more refined per-iteration search. It should be noted that the FE-based protocol is not claimed to be inherently unfair; rather, this study demonstrates that its fairness assumption holds only for algorithms with comparable per-iteration costs. Where this assumption fails, multi-protocol assessment becomes necessary.

## 6. Conclusions

This study evaluated five metaheuristic algorithms (PFA, LSHADE-CnEpSin, LSHADE-SPACMA, MadDE, and BO) on frequency-constrained truss optimization using two complementary protocols: (i) standardized FE ceiling (MaxFEs = 10,000 × D) and (ii) extended budget (MaxFEs = 20,000 × D) to assess scalability and protocol sensitivity across five problems (10-, 37-, 52-, 72-, and 200-bar trusses).

Key findings:

• Under the 10,000 × D protocol, LSHADE variants dominated (ranks 1.33–2.00), consistently reaching known optima with minimal variance, while PFA ranked lowest (4.33–5.00) despite fastest execution times, exhibiting best-cost gaps of 0.06–0.7% due to premature termination from non-uniform FE allocation (62–111 iterations vs. 2000+ for DE methods).
• Extended budget evaluation (20,000 × D) revealed systematic protocol dependence; PFA’s performance gaps reduced by 44–79% with rankings improving to 2nd–3rd, while LSHADE variants showed negligible change (<0.001 kg), confirming saturation under lower budgets. On larger problems, ranking patterns shifted substantially—BO dominated 72-bar (328.24 kg), while PFA achieved competitive placements (rank 2–3) across all scales.
• Protocol dependence validated: FE ceilings favor uniform-allocation methods (LSHADE and MadDE) by maximizing iteration counts; extended budgets partially mitigate bias (44–79% gap reduction).

The cumulative evidence reveals that algorithmic rankings in TPFC optimization are not absolute but protocol-dependent artifacts: FE ceilings systematically favor uniform-allocation methods, extended budgets narrow performance gaps, and iteration-based termination exposes intrinsic convergence quality. Robust benchmarking requires multi-protocol evaluation—reporting results under complementary termination criteria prevents algorithmic merit with resource-allocation compatibility. The 10,000 × D standard, while ensuring equal computational budgets, may inadvertently penalize exploration-intensive strategies by implicitly assuming uniform per-iteration FE consumption across competing methods; therefore, combining it with extended-budget assessments enables a more complete characterization of algorithms. Several limitations should be noted. The analysis is limited to five benchmark truss problems under deterministic conditions, so direct generalization to real-world systems with uncertainties may be limited. The dual protocol framework considers only two FE budget levels, and a broader sensitivity analysis could provide deeper insight. In addition, the penalty parameter was adopted from reference benchmarks without further investigation. These points suggest directions for future work, including validation on larger-scale problems under uncertainty, multi-objective extensions, and improved constraint handling strategies. Future research should pursue: (i) scalability validation on industrial-scale problems (600-bar and 1410-bar dome trusses) to confirm trends observed in 72-bar and 200-bar benchmarks and (ii) multi-objective extensions for Pareto-based frequency-constrained optimization, advancing structural dynamics applications beyond single-objective frameworks.

From a practical engineering perspective, the results of this study provide actionable guidance for algorithm selection in TPFC problems. When the computational budget is severely limited (MaxFEs = 10,000 × D), DE-based methods such as LSHADE-CnEpSin and MadDE are recommended for their uniform FE allocation and consistent convergence. When an adequate budget is available (MaxFEs = 20,000 × D), exploration-intensive methods such as PFA and BO become competitive, particularly for intermediate-scale problems such as the 72-bar truss, where ranking reversals were observed. For large-scale problems such as the 200-bar truss, distinctions among top-tier methods become marginal under extended budgets, suggesting that computational cost rather than algorithm selection becomes the dominant practical consideration.

## Figures and Tables

**Table 1:** Design variable groups and bounds for the 37-bar truss.

Variable Type	Variables	Lower Bound	Upper Bound
Cross-sectional areas	A_1_–A_14_ (grouped symmetrically)	1 × 10^−4^ m^2^ (1 cm^2^)	10 × 10^−4^ m^2^ (10 cm^2^)
Y-coordinates	Y_3_ = Y_19_, Y_5_ = Y_17_, Y_7_ = Y_15_, Y_9_ = Y_13_, Y_11_	0.3 m	3.0 m

**Table 2:** Material properties and frequency constraints for the 37-bar truss.

Property	Value
Modulus of elasticity (E)	210 GPa
Material density (ρ)	7800 kg/m^3^
Added mass	10 kg at nodes 2, 4, 6, 8, 10, 12, 14, 16, 18
Frequency constraints	$f_{1}$ ≥ 20 Hz, $f_{2}$ ≥ 40 Hz, $f_{3}$ ≥ 60 Hz

**Table 3:** Material properties and design parameters.

Property/Unit	Value
Modulus of elasticity (E)	210 GPa
Material density (ρ)	7800 kg/m^3^
Added mass	50 kg
Cross-sectional area bounds	0.1–100 mm^2^
Frequency constraints	ω_1_ ≤ 15.916 Hz, ω_2_ ≥ 28.648 Hz

**Table 4:** Material properties, constraints, and loading conditions.

Item	72-Bar Spatial Truss	200-Bar Planar Truss
Elastic modulus (E)	68,947.6 MPa (10,000 ksi)	206,842.7 MPa (30,000 ksi)
Material density	27.801 kg/m^3^ (0.1 lb/in^3^)	78.678 kN/m^3^ (0.283 lb/in^3^)
Area bounds	0.645–129.032 cm^2^	0.645–129.032 cm^2^
Stress limits	±172.369 MPa	±68.947 MPa
Displacement limits	±0.635 cm	–
Loading conditions	Concentrated loads at top node	Three load cases (horizontal, vertical, combined)

**Table 5:** Comprehensive performance metrics for five metaheuristic algorithms across three TPFC benchmark problems under the standardized 10,000 × D FE protocol.

Problem	Algorithm	Best	Mean	Std	Median	Avg_Iter	Time (s)	Best Rank	Avg_Rank
10-Bar Truss	MadDE	552.0422	552.4942	0.515641	552.1553	1996	16.61	2	1.67
10-Bar Truss	LSHADE-CnEpSin	552.042	555.466	0.646682	555.584	2163	17.07	1	2
10-Bar Truss	LSHADE-SPACMA	555.584	555.5841	0.00013	555.584	2163	17.13	5	3
10-Bar Truss	BO	552.057	556.403	3.395984	555.9569	3333	21.73	3	4
10-Bar Truss	PFA	552.4025	557.3582	3.054682	557.6523	62	16.03	4	4.33
37-Bar Truss	LSHADE-CnEpSin	361.8358	361.8358	0	361.8358	2501	145.11	2	1.33
37-Bar Truss	LSHADE-SPACMA	361.8358	361.8358	0	361.8358	2501	147.57	1	1.67
37-Bar Truss	MadDE	361.8468	361.8815	0.020664	361.8752	1373	142.4	3	3
37-Bar Truss	BO	361.9885	362.6107	0.79565	362.3218	6333	154.58	4	4.33
37-Bar Truss	PFA	362.1815	362.8512	0.652551	362.7187	110.8	138.65	5	4.67
52-Bar Truss	MadDE	200.919	201.3533	0.403851	201.1387	1726	121.84	3	1.67
52-Bar Truss	LSHADE-CnEpSin	200.9163	202.4975	3.216507	200.9163	2300	120.93	2	2
52-Bar Truss	LSHADE-SPACMA	200.9163	203.2881	3.684972	200.9163	2300	122.06	1	2.33
52-Bar Truss	BO	200.9806	205.7122	3.75661	208.8262	4333	128.63	4	4
52-Bar Truss	PFA	202.3339	209.2449	5.011288	209.2572	82.6	114.71	5	5

**Table 6:** Computational budget sensitivity analysis across five TPFC benchmarks.

Problem	Budget	Algorithm	Best (kg)	Mean (kg)	Std (kg)	Avg Iter	Gap †	Rank
10-bar	10,000 × D	LSHADE-CnEpSin	552.0420 *	555.466	0.647	2163	—	1
(D = 10)		MadDE	552.0422 *	552.494	0.516	1996	+0.0004%	2
		BO	552.1018 *	556.820	2.950	2003	+0.011%	3
		PFA	552.4025 *	552.656	0.351	62	+0.065%	4
		LSHADE-SPACMA	561.7458 *	568.200	4.114	2004	+1.76%	5
	20,000 × D	LSHADE-CnEpSin	552.0423	555.068	1.154	4323	—	1
		BO	552.1018	556.820	2.950	4012	+0.011%	2
		PFA	552.2397	555.229	2.271	124	+0.036%	3
		MadDE	553.0249	553.825	0.495	3989	+0.178%	4
		LSHADE-SPACMA	561.7458	568.200	4.114	4001	+1.76%	5
	Change	LSHADE-CnEpSin	+0.0003	−0.398	+0.507	—	stable	—
		PFA	−0.1628	+2.573	+1.920	—	−44%	+1
37-bar	10,000 × D	LSHADE-CnEpSin	361.8358 *	361.836	0.000	2501	—	1
(D = 19)		LSHADE-SPACMA	361.8358 *	368.318	3.034	2500	0%	2
		MadDE	362.3594 *	362.489	0.082	2499	+0.145%	3
		BO	362.1134 *	363.183	1.382	2500	+0.077%	4
		PFA	362.1815 *	362.418	0.220	111	+0.095%	5
	20,000 × D	LSHADE-CnEpSin	361.8358	361.836	0.000	5002	—	1
		PFA	361.9079	362.748	0.607	221	+0.020%	2
		BO	361.8917	362.325	0.714	5001	+0.015%	3
		MadDE	362.5474	362.730	0.102	4998	+0.196%	4
		LSHADE-SPACMA	364.8792	371.882	3.254	4999	+0.841%	5
	Change	LSHADE-CnEpSin	0.0000	0.000	0.000	—	stable	—
		PFA	−0.2736	+0.330	+0.387	—	−79%	+3
52-bar	10,000 × D	LSHADE-CnEpSin	200.9163 *	200.916	0.000	2146	—	1
(D = 13)		MadDE	200.9190 *	205.185	1.858	2145	+0.001%	2
		BO	200.9526 *	203.987	3.536	2144	+0.018%	3
		LSHADE-SPACMA	217.4998 *	254.468	22.201	2145	+8.25%	4
		PFA	202.3339 *	206.420	3.771	83	+0.705%	5
	20,000 × D	LSHADE-CnEpSin	200.9163	200.916	0.000	4289	—	1
		PFA	201.2942	206.410	3.777	166	+0.188%	2
		BO	200.9526	204.298	3.794	4287	+0.018%	3
		MadDE	202.7304	205.922	1.492	4285	+0.903%	4
		LSHADE-SPACMA	217.4998	254.468	22.201	4286	+8.25%	5
	Change	LSHADE-CnEpSin	0.0000	0.000	0.000	—	stable	—
		PFA	−1.0397	−0.010	+0.006	—	−73%	+3
72-bar	20,000 × D ‡	BO	328.2367	328.386	0.300	—	—	1
(D = 4)		PFA	341.0286	386.477	19.362	—	+3.90%	2
		MadDE	354.6196	380.409	12.238	—	+8.04%	3
		LSHADE-CnEpSin	407.9192	444.157	16.585	—	+24.3%	4
		LSHADE-SPACMA	414.5576	479.246	28.255	—	+26.3%	5
200-bar	20,000 × D ‡	LSHADE-CnEpSin	2174.333	2174.333	0.000	—	—	1
(D = 29)		BO	2174.386	2174.802	0.448	—	+0.002%	2
		PFA	2174.596	2174.890	0.301	—	+0.012%	3
		MadDE	2287.212	2312.891	13.537	—	+5.19%	4
		LSHADE-SPACMA	2632.360	3826.459	440.327	—	+21.1%	5

## Data Availability

Data available upon request.
